# Supplementary material for: New austrolimulid from Russia supports role of Early Triassic horseshoe crabs as opportunistic taxa
Source: PeerJ. 2021 Jun 30;9:e11709. doi: 10.7717/peerj.11709 (PMC8254475; doi:10.7717/peerj.11709)
Supplement: Supplemental Information 3 [file peerj-09-11709-s003.docx]

| **Landmark number** | **Description of landmark** |
| --- | --- |
| Landmark 1 | Anterior-most prosomal point along sagittal line |
| Landmark 2 | Distal-most prosomal point along sagittal line |
| Landmark 3 | Posterior-most point of ophthalmic ridge |
| Landmark 4 | Distal-most point of genal spine |

**Supplementary Table 1:** Summary of digitised landmarks depicted in Figure 2.
